# Prion-Like Mechanisms in Parkinson’s Disease

**DOI:** 10.3389/fnins.2019.00552

**Published:** 2019-06-18

**Authors:** Jiangnan Ma, Jing Gao, Jing Wang, Anmu Xie

**Affiliations:** Department of Neurology, Affiliated Hospital of Qingdao University, Qingdao, China

**Keywords:** Parkinson’s disease, prion-like mechanisms, α-synuclein, templated conformational change, transcellular propagation

## Abstract

Formation and aggregation of misfolded proteins in the central nervous system (CNS) is a key hallmark of several age-related neurodegenerative diseases, including Parkinson’s disease (PD), Alzheimer’s disease (AD), and amyotrophic lateral sclerosis (ALS). These diseases share key biophysical and biochemical characteristics with prion diseases. It is believed that PD is characterized by abnormal protein aggregation, mainly that of α-synuclein (α-syn). Of particular importance, there is growing evidence indicating that abnormal α-syn can spread to neighboring brain regions and cause aggregation of endogenous α-syn in these regions as seeds, in a “prion-like” manner. Abundant studies *in vitro* and *in vivo* have shown that α-syn goes through a templated conformational change, propagates from the original region to neighboring regions, and eventually cause neuron degeneration in the substantia nigra and striatum. The objective of this review is to summarize the mechanisms involved in the aggregation of abnormal intracellular α-syn and its subsequent cell-to-cell transmission. According to these findings, we look forward to effective therapeutic perspectives that can block the progression of neurodegenerative diseases.

## Introduction

Parkinson’s disease (PD) is the most prevalent neurodegenerative motor disorder worldwide, characterized by motor symptoms, such as bradykinesia, resting tremor, rigidity, and postural instability. However, a myriad of non-motor symptoms, such as constipation, dementia, anosmia, depression, and sleep disturbances, regularly manifest prior to the motor symptoms and affect quality of life (Dehay and Fernagut, 2016; Tyson et al., 2016). The primary pathological manifestation of PD is the relative selective loss of dopaminergic neurons in the substantia nigra pars compacta (SN), which results in a significant decrease in the dopamine (DA) content of the striatum. The exact cause of this pathological change is still unclear. Genetic and environmental factors, aging, and oxidative stress may be involved in the degenerative death of dopaminergic neurons. Lewy bodies (LBs) and Lewy neurites (LNs) are the morphological markers of neuron degeneration. LBs, which are intraneuronal proteinaceous cytoplasmic inclusions, are composed primarily of misfolded α-syn (Sian-Hulsmann et al., 2015). The degeneration of dopaminergic neurons underlie the motor symptoms of PD, however, the causes of cell degeneration and the occurrence of more complicated non-motor symptoms have attracted more and more attention. Not only that, many observations support the “prion-like” behavior of α-syn, which postulates that misfolded α-syn is capable of transferring between interconnected neuron networks and acting as a template to induce further aggregation of intracellularly transferred α-syn.

## Prion Diseases and Prion-Like Mechanism

Prion diseases are fatal transmissible neurodegenerative disorders with genetic, sporadic, and acquired forms (Acquatella-Tran Van Ba et al., 2013). Prions are the infectious agents responsible for the transmissible spongiform encephalopathies (TSEs), a group of lethal neurodegenerative diseases (Le et al., 2015). These diseases also include Kuru, Creutzfeldt-Jakob disease (CJD), Gerstmann–Sträussler–Scheinker disease (GSS), and fatal familial insomnia in human, bovine spongiform encephalopathy (BSE) in cattle, and scrapie in sheep (Goedert et al., 2010; Costanzo and Zurzolo, 2013; Tamguney and Korczyn, 2018).

**FIGURE 1:**
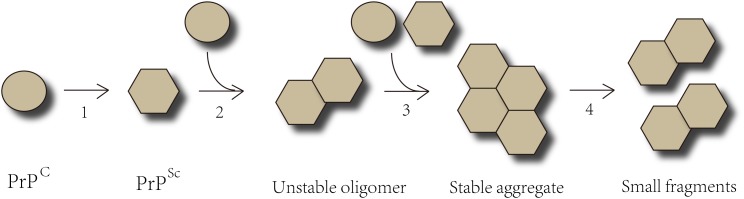
The Templated Conformation Change of Prions. Step 1- The α-helices of PrP^C^ transform into the β-sheets conformation to form pathological PrP^Sc^. Step 2- PrP^Sc^ interacts with PrP^C^ and converts them into pathological form. Step 3- PrP^Sc^ binds to the cognate prion molecules. Unstable oligomeric species grow by recruiting additional unfolded or oligomeric species of the same protein until forming a stable nucleus. Step 4- The prion aggregates break into small fragments that act as seeds and spread indefinitely from the point of infection to the CNS.

Prion protein (PrP), is capable of resisting chemical therapy or physical prevention that inhibits ordinary infectious agents. PrP is encoded by the endogenous gene-*PRNP* and expressed in a variety of cells whether infected or not. PrP^C^, a glycosylphosphatidylinositol (GPI) anchored protease-sensitive protein of 33–35 kDa, is the normal product of *PRNP* gene (Le et al., 2015; Quek and Hill, 2017). PrP^C^ is commonly detected on the surface of cell membranes and dominantly located in neurons, though it is ubiquitously expressed. However, the specific biological functions of PrP^C^ are still unknown. It is believed that PrP^C^ participates in many physiological processes, including signal transduction, maintaining copper or zinc homeostasis, and acting as a receptor (Halliday et al., 2014; Quek and Hill, 2017; Tamguney and Korczyn, 2018). The crucial role of copper-binding sites in maintaining the neuritogenesis function in PrP^C^ has been proven (Nguyen et al., 2019). The central causative event in neurodegeneration is the conversion of the normal form PrP^C^ into a protease-resistant, disease-associated form PrP^Sc^, which is known as templated conformation change. These two isoforms of PrP share an identical composition of polypeptide chain but differ in secondary and tertiary structure. The α-helices of native PrP^C^ transform into β-sheet conformation to form the pathological PrP^Sc^. The configuration of PrP^Sc^ is quite stable and equipped to interact with molecules in a similar state. The misfolded protein can proliferate via templated conformational change. Exogenous PrP^Sc^ interacts with endogenous PrP^C^ and induces it to pathological conformational transition. The unstable oligomeric species grow by recruiting and integrating PrP^C^ and PrP^Sc^ constantly until forming stable prion aggregates (Acquatella-Tran Van Ba et al., 2013; Renner and Melki, 2014). PrP^Sc^ aggregates result in cell rupture, and shed PrP^Sc^ acts as seeds which propagate into other cells indefinitely (Figure 1). Both PrP^C^ and PrP^Sc^ are detected in exosomes from diverse sources including neuronal cells, blood, and cerebrospinal fluid (CSF). (Yin et al., 2014; Quek and Hill, 2017) Coupled application of immunogold labeling and electron microscopy imaging confirmed the presence of PrP on exosome membranes, showing that exosomes may play a crucial role in transferring prion infectivity. Beyond that, it has been verified that exosomes are able to spread along tunneling nanotubes (TNTs), suggesting a function in cell-to-cell propagation of infectious prions (Costanzo and Zurzolo, 2013; Kaufman and Diamond, 2013; Yin et al., 2014). Propagating prions cause devastating neurodegeneration cell by cell, but it is still under debate how infectious prions induce TSEs and eventually neuronal death.

Emerging research highlights that many neurodegenerative diseases share key prion-like similarities with the progress of prion diseases. α-Syn is a typical pathogenic agent for PD and exhibits properties of self-aggregation and propagation, just like prions do.

In addition to the templated misfolding, the transmission between cells is also a central feature of prions. Under normal conditions, infection of prions gives rise to dissemination through the peripheral and central nervous system (CNS) by distal neuronal spreading. Prions induce epidemics due to the transmission between individuals and species (Goedert et al., 2010). However, there is no epidemiological data to show that PD is infectious. Therefore, an extended definition of “seeding” is used to describe the transmissibility of misfolded proteins in neurodegenerative diseases *in vivo* (Fernandez-Borges et al., 2013; Halliday et al., 2014; Diack et al., 2016).

## The Prion-Like Mechanism of PD

### Basic α-Synuclein Biology

α-Synuclein is a 14 kDa protein composed of 140 amino acids and encoded by *SNCA* (the coding gene) (Tamguney and Korczyn, 2018). High concentrations of α-syn are present in presynaptic terminals, where it associates with vesicular membranes (Volpicelli-Daley et al., 2011; Narkiewicz et al., 2014; Renner and Melki, 2014). Abundant evidence suggests that the normal physiological functions of α-syn include direct interaction with cell membrane phospholipids (especially referring to vesicles), neurotransmitter release, and enhancement of microtubule formation (Recasens and Dehay, 2014; Hasegawa et al., 2016; Burre et al., 2018). A study by Cali et al., using organelle-targeting Ca^2+^-sensitive aequorin probes, demonstrates that physiological levels of α-syn are required to maintain normal mitochondrial function and morphological integrity. Consequently, α-syn overexpression and/or changes in its aggregation properties induce the redistribution of α-syn and the loss of modulation on mitochondrial function (Cali et al., 2012). In addition, the accumulation of α-syn can impair synaptic dopamine release and cause the death of nigrostriatal neurons (Longhena et al., 2017). Insights into the localization and function of α-syn in subcellular compartments would facilitate the understanding of the pathology of α-syn.

Accumulation of oligomers and larger aggregates of misfolded α-syn is detected in multiple neurodegenerative diseases called synucleinopathies, including Parkinson’s disease (PD), dementia with Lewy bodies (DLB), and multiple system atrophy (MSA) (Tamguney and Korczyn, 2018). The initial pathogenesis of synucleinopathies is still considered involving the conversion of soluble α-syn into insoluble aggregates via spontaneous nucleation. This pathological conversion could be caused by countless environmental or genetic factors, and is quite likely the result of interaction among diverse factors (Karpowicz et al., 2019).

α-syn is composed of three domains: an amino terminus with α-helix, a non-amyloid component (NAC), and an unstructured carboxy terminus. Soluble α-syn is natively unstructured and monomeric. These three domains are essential for the pathogenic progress observed in PD and other synucleinopathies. The amino terminus of α-syn forms an α-helical structure upon binding to protein interactors or lipid membranes (Kim et al., 2014; Narkiewicz et al., 2014; Lawand et al., 2015). Accumulating biophysical and biochemical studies have demonstrated that interactions between α-syn and lipids influence α-syn oligomerization and aggregation, both *in vitro* and *in vivo*. Mainly three kinds of lipids are associated with pathological interactions with α-syn, i.e., fatty acids, sterols and sphingolipids. Both the chemical properties of lipids and the lipid-to-protein-ratio could modulate the aggregation propensity of α-syn. Lipid vesicles interacting with monomeric and fibrillar α-syn are possible to affect the initiation of the amyloid formation and the amplification of the toxic aggregates. Measurements of the kinetics of amyloid formation *in vitro* in the presence of different lipid systems allow for the study of mechanistic details of the effects of lipids on α-syn aggregation and toxicity, and open a new therapeutic road to attenuate or prevent crucial events (Galvagnion, 2017; Suzuki et al., 2018).

A technique called cryo-electron microscopy (cryo-EM) was applied to capture the structure of full-length α-syn fibril. Two protofilaments intertwining along an approximate 21 screw axis into a left-handed helix form a polar fibril composed of stacked β-strands. The backbone of residues 38–95, including the fibril core and the NACore, is well illustrated in the EM map. Residues 50–57, containing three mutation sites linked to familial PD, form the interface between the two protofilaments and are associated with fibril stability. A hydrophobic cleft at one end of the fibril may have an impact on fibril elongation, and has implications for diagnosis and treatment of PD (Dillard et al., 2018; Guerrero-Ferreira et al., 2018; Li et al., 2018a,b).

α-Syn turns into oligomeric and/or fibrillar conformations in particular pathological conditions, including gene mutations of *SNCA*, decreased rate of clearance, possible alteration of α-syn (such as truncations), oxidative stress, iron concentration, or posttranslational modifications (Lawand et al., 2015; Sian-Hulsmann et al., 2015). In particular, posttranslational modifications of α-syn, such as phosphorylation, ubiquitination, acetylation, sumoylation, and nitration, have been observed to alter the structure and function of α-syn, and are related to α-syn aggregation and neurotoxicity (Hodara et al., 2004; Kim et al., 2014; Hasegawa et al., 2017). Most cases of PD are sporadic, and rare familial PD is induced by missense mutations of *SNCA* or multiplications of the gene. Other genetic mutations, including *PARK-LRRK2* and *PARK-VPS35*, are also related to the pathogenesis of α-syn (Hyun et al., 2013; Chan et al., 2017). The N-terminal region of α-syn contains pathogenic gene mutation sites A53T, A53E, A30P, E46K, H50Q, and G51D, and six imperfectly conserved repeats (KTKEGV) that promote protein interaction (Hasegawa et al., 2016; Rey et al., 2016; Burre et al., 2018). Recent studies with animal and cell models, as well as autopsy studies of PD patients, provide abundant evidences for “prion-like” behavior of α-syn.

### Evidence for the Prion-Like Properties of α-Synuclein

#### Abnormal Aggregation of α-Syn

The primary component of the proteinaceous filaments of LBs and LNs is α-syn. A slight accumulation of LBs or LNs is capable of reducing striatal tyrosine hydroxylase (TH) levels, which implies the preclinical prodromal phase of PD (Chan et al., 2017). Because dopamine neurons are invariably compromised in PD, Perez and Hastings have been exploring the functions of α-syn with particular relevance to dopamine neurons. TH, the rate limiting enzyme in dopamine synthesis, converts tyrosine to L-dihydroxyphenylalanine (L-DOPA), which is then converted into dopamine by aromatic amino acid decarboxylase (AADC). In several rodent models overexpressing α-syn, reduced TH activity and/or diminished dopamine synthesis are observed. It was also confirmed that α-syn significantly reduced AADC activity and phosphorylation in cells (Perez et al., 2002; Peng et al., 2005; Tehranian et al., 2006).

*In vitro* studies indicate that recombinant α-syn can polymerize into amyloidogenic fibrils with similar morphologies and staining as those obtained from extracts of disease-affected brains (Narkiewicz et al., 2014). *In vitro* aggregation assays are feasible tools for studying the mechanisms responsible for the formation of different α-syn species, their structural properties and functions, as well as for the development of new drug capable of restraining from the aggregation of α-syn. However, recombinant preformed α-syn fibrils (PFFs) are not naturally toxic to cultured neurons, as neurons in the absence of α-syn (α-syn KO) display no toxicity following transduction with the same concentration of α-syn PFFs that could produce robust pathologic α-syn inclusions and cause eventual cell death in wild type (WT) neurons (Volpicelli-Daley et al., 2011).

When α-syn is incubated *in vitro* at a high concentration under shaking conditions, it undergoes a conformational change and turns into fibrils within a few days. In contrast, little or no fibrils form at a lower α-syn concentration without shaking, and the protein needs more time to assemble. However, the number of fibrils would increase upon adding preformed fibrils into the monomer, suggesting that the conformational conversion of α-syn is facilitated by accretion of misfolded α-syn. In cultured cell models, when preformed α-syn fibrils are added by lipofection, a large number of phosphorylated and partially ubiquitinated α-syn aggregations similar to LBs, both morphologically and biochemically, are detected in few days. Cultured cells with α-syn aggregates show signs of slow degeneration, with a noticeable impairment of proteasome activity, which resembles disease progression in patients with PD (Hasegawa et al., 2017). These studies indicate that the mechanism underlying the pathological aggregation of α-syn is as follows: exogenous abnormal α-syn acts on the endogenous natural α-syn of recipient cells and causes pathological aggregation of α-syn.

It was observed that treating SH-SY5Y cell lines with recombinant human α-syn short amyloid fibrils resulted in sustained aggregation and accumulation of endogenous α-syn. This approach provides an excellent tool for potential therapeutic screening of pathogenic α-syn aggregates in cell culture (Aulic et al., 2014). Peelaerts et al. injected structurally well-defined human α-syn strains (including oligomers, ribbons, and fibrils) into mice brain, then reported that distinct α-syn strains show differential seeding capacities, inducing strain-specific pathology and neurotoxic phenotypes. These results point to α-syn short amyloid fibrils as the pathological species of α-syn aggregates involved in the transmission of α-syn pathology (Peelaerts et al., 2015; Braak and Del Tredici, 2016; Hasegawa et al., 2017). Tarutani et al. examined the seeding properties of various forms of α-syn *in vitro*, cells, and mice experimental models, and eventually, a consistent conclusion with Peelaerts was reached (Tarutani et al., 2016).

Several studies have reported that α-syn aggregation is induced by environmental and other exogenous factors, including pesticides, herbicides, heavy metals, polycations, histones, organic solvents, heat shock proteins (Hsp) and some small chemical compounds (Uversky, 2007; Narkiewicz et al., 2014). Recently, some studies, that have characterized the microscopic process in the mechanism of aggregation of α-syn, show that solution conditions may play an important role. For example, at faintly acidic pH values (such as those present intracellularly, including within endosomes and lysosomes), the expansion of α-syn aggregation is faster than at normal physiological pH values (Buell et al., 2014). Additional factors destabilizing the native fold of α-syn, such as point mutations, denaturants, and higher temperature, have been shown to enhance fibril formation (Rochet and Lansbury, 2000; Narkiewicz et al., 2014). The factors responsible for the kinetics of α-syn fibrillization include initial protein concentration, molecular crowding, agitation, pH, temperature, and ionic strength (Narkiewicz et al., 2014). These findings provide new directions for the prevention and treatment of PD, but these ideas need further development.

Under physiological conditions, normal nerve cells, for the lifetime of an individual, are capable of degrading and eliminating errant proteins and/or toxic metabolites. Intracellular homeostasis of α-syn depends on proper degradation mechanisms. Autophagic impairment has been reported in a donor recipient co-culture cell model to increase intercellular transfer of α-syn. Although, it is unknown whether if impaired autophagy in donor cells leads to increased release, or increased deposition and retention of transferred α-syn (Lee et al., 2013). There are three pathways currently involved in PD: chaperone-mediated autophagy, microautophagy, and macroautophagy (Karabiyik et al., 2017). The first process is carried out through the ubiquitination of the misfolded α-syn and their subsequent treatment by chaperones. When the function of the chaperones is altered, this option cannot be performed (Van Bulck et al., 2019). The other two autophagy methods are also altered in most neurodegenerative diseases for various reasons including the biogenesis of autophagosomes or lysosomal function (Menzies et al., 2017). Exosomal secretion of α-syn aggregates has been observed in response to knockdown of macroautophagy component ATG5 (Fussi et al., 2018). It has been demonstrated, by utilizing cell models, that α-syn inclusions cannot be effectively degraded and can impair overall macroautophagy by reducing autophagosome clearance, which may contribute to increased cell death (Tanik et al., 2013). In brief, perturbations in these degradation pathways may create an environment that leads to the formation and propagation of misfolded α-syn (Lee et al., 2010; Tanik et al., 2013; Lopes da Fonseca et al., 2015; Victoria and Zurzolo, 2015).

#### Transcellular Propagation of α-Synuclein

Braak and colleagues first revealed that LBs and LNs are enriched in the dorsal motor nucleus of the vagal nerve (dmX) and olfactory bulb prior to the appearance of classic motor symptoms in PD by post-mortem histopathological studies; afterward, they are present in interconnected brain regions with the development of disease (Braak et al., 2003; Braak and Del Tredici, 2016). They describe the assumption of active retrograde transport of α-syn for stages 1–6. In stage 1, α-syn is present within the olfactory bulb, dmX, and the intermediate reticular zone. In this stage, loss of smell is reported in patients. In stage 2, the level-setting nuclei of the lower brainstem are involved, followed by a group of non-thalamic nuclei that project widely to the cerebral cortex. In stage 2, patients may suffer from disturbance of sleep and wakefulness, movement problems, lowered blood pressure, constipation, and emotional disorders. In stage 3, the α-syn pathology reaches the midbrain nuclei (including substantia nigra pars compacta) and the central subnucleus of the amygdala. Patients in this stage manifest thermoregulation disorder, and cognitive impairment. In stage 4, Lewy pathology invades additional subnuclei of the amygdala and the cerebral cortex. Clinically, four major symptoms, these being bradykinesia, resting tremor, rigidity, and postural instability, can be observed. During stages 5 and 6, the deep layers of the neocortex are involved, and patients may suffer motion fluctuations, frequent fatigue, visual hallucination, dementia, and psychiatric symptoms (Goedert et al., 2010, 2014; Dunning et al., 2012; Braak and Del Tredici, 2016). The former three stages compose the pre-symptomatic phase, and the latter stages the symptomatic phase (Braak et al., 2003). This pathological propagation describes the symptomatic progression of patients with PD. These observations provide the basis for the prion-like hypothesis of α-syn.

##### The propagation of PD among different organs

Braak et al. argue that the olfactory bulb and enteric plexus of the stomach may be starting points of pathology in many neurodegenerative diseases including PD (Acquatella-Tran Van Ba et al., 2013; Renner and Melki, 2014; Rey et al., 2018). The presence of Lewy pathology in the enteric nervous system (ENS) led to the hypothesis that α-syn pathology starts in the peripheral nervous system (PNS) with α-syn serving as a direct seed (Goedert et al., 2014). In order to test this hypothesis, Manfredsson et al. used two distinct models of rodents and non-human primates (NHP): the α-syn viral overexpression model and the preformed fibril (PFF) model. Their data suggest that α-syn can be transported from ENS to the CNS brainstem in both rodents and NHPs, however, the pathology was neither sustained, nor did it spread (Manfredsson et al., 2018).

A recent study by Takahashi and colleagues indicates that α-syn PFF inoculation into the mouse gastrointestinal tract induces α-syn pathology resembling that at early stage of PD, which supports the “gut-to-brain” hypothesis (Uemura et al., 2018). According to Braak’s hypothesis, abnormal α-syn is spread between anatomically interconnected systems (Recasens and Dehay, 2014; Braak and Del Tredici, 2016). However, this hypothesis is merely confined to snapshots of degeneration mechanisms in independent post-mortem PD patients after diagnosis. A separate, alternative hypothesis proposed by Engelender and Isacson is that selective vulnerability underlies pathogenesis, which better accounts for the current neurobiology of PD symptoms progression compared to the hypothesis proposed by Braak that the pathology ascends from the PNS to the CNS. According to this assumption, less-resistant nerve cells suffer attack earlier; conversely, more-resistant cells can survive by benefiting from cellular compensation. The “threshold theory” explains that signs from the PNS and brainstem appear earlier than motor signs. The symptoms of PD only show signs when the functional reserve of neurons (and their connecting brain regions) is undercompensated. In conclusion, early symptoms of PD reflect loss of function in the least compensated tissues, such as the GI tract, olfactory system, and brainstem (Engelender and Isacson, 2017).

Svensson et al. used data from the Danish National Patient Registry (DNPR) and reported that patients who had undergone full truncal vagotomy had a clear reduced risk for PD compared to both super-selective vagotomies and the general population, suggesting that the vagal nerve was critically involved in the pathogenesis of PD (Svensson et al., 2015; Tysnes et al., 2015). Holmqvist et al. (2014) provided the first experimental evidence with injecting brain lysate containing different α-syn forms (monomeric, oligomeric, and fibrillar) and distinct recombinant α-syn into the intestinal wall. They demonstrated that different α-syn forms spread from the gut to the brain via the vagal nerve in a time-dependent manner (Holmqvist et al., 2014). In addition, α-syn is particularly abundant in the Appendix, which makes it an anatomical candidate for the initiation of PD pathology. Two independent epidemiological datasets showed that appendectomy decades before PD onset was associated with a reduced risk for PD and delayed the age of PD onset. Possible mechanisms were studied when it was observed that Appendix lysates induce the rapid cleavage and oligomerization of full-length α-syn (Killinger et al., 2018). The Appendix has been recently shown to contain an abundance of α-syn and PD pathology-associated α-syn truncation products that accumulate in LBs in neurologically intact individuals. α-syn is detected in the whole ENS of healthy people and PD patients, suggesting that the discovery of α-syn alone is not sufficient to explain the progress of PD. The removal of the Appendix is possibly not sufficient to inhibit the exposure of CNS to α-syn via vagal retrograde transport. However, whether the Appendix, as a GI tract lymphoid organ, has an enhanced capacity to generate truncated α-syn amylogenic seeds is unknown. Further studies are required to elucidate the role of Appendix in PD (Marras et al., 2016; Svensson et al., 2016; Yilmaz et al., 2017; Palacios et al., 2018).

Experiments *in vivo* by DiMonte were carried out to clarify the relation between α-syn accumulation in the brain and in peripheral organs, and to explore potential mechanisms involved in long-distance transmission of α-syn. These studies revealed a route-specific transmission of α-syn from the brain to the stomach of rats, following targeted midbrain overexpression of human α-syn. A specific tendency of vagal motor neurons and efferent fibers to accrue α-syn and transfer it to peripheral organs has also been studied. The significant work by DiMonte suggests a potential for two-way spread between the ENS and CNS (Ulusoy et al., 2017). Another study by DiMonte emphasized that rapid long-distance diffusion and accumulation of monomeric and oligomeric α-syn might result in a fatal neuronal burden and contribute to the development of PD. However, the field of α-syn staining in peripheral tissues is not mature right now, hence we have insufficient knowledge about the pathology in this area (Helwig et al., 2016; Ulusoy et al., 2017; Borghammer, 2018).

##### The host-to-graft transmission of PD

The first tentative evidence for the transcellular propagation of α-syn was in the autopsy of patients with PD who had had embryonic brain tissue transplanted into the striatum to replace degenerated dopaminergic neurons (Kordower et al., 2008; Hansen et al., 2011). Over 10–16 years after injection of the graft, when examining the brain tissues of these patients post-mortem, it was found that pathologic α-syn inclusions, similar to those in the host brain, appeared in the cells generated from the grafted embryonic tissue (Kaufman and Diamond, 2013; Goedert et al., 2014). One possibility is that pathological α-syn spread from the degenerated host brain tissue to the healthy young brain tissue, recruited further α-syn from the recipient cells and generated α-syn inclusions. Another study found that patients with PD who survived for only 4–9 years after transplantation did not manifest pathological progress. These studies indicate that the prion-like pathological process develops at a slow rate (Kordower et al., 2008).

Consequently, numerous research programs were launched to explore the “host-to-graft transmission” hypothesis in animal models, such as mice and NHP. Seven distinct experimental categories have been used to investigate the spreading of α-syn:

(1) Mougenot and colleagues extracted insoluble α-syn aggregates from the cerebral homogenate of gerontic pathogenetic mice (12–18 months) overexpressing α-syn or expressing mutated A53T α-syn and injected the extract into the brain of younger transgenic mice (7–9 weeks). They found that α-syn aggregation appeared in the brain of the young mice, who showed early symptoms of PD. In grafts, LBs could be observed in up to 5% dopaminergic neurons, similar to the proportion of LB-containing neurons in the substantia nigra of patients with PD patients (Mougenot et al., 2012). On the contrary, after injection of identical extract to mice with *SNCA* gene knockout, the pathologic progress and PD manifestations could not be observed (Luk et al., 2012b; Recasens et al., 2014; Fernandez-Borges et al., 2015; Peelaerts et al., 2015).
(2) When synthetic α-syn PFF were injected into the striatum or substantia nigra of rat brain over time, α-syn inclusions spreading over the brain were observed by immunohistochemistry, while mice injected with soluble α-syn did not exhibit pathological symptoms, suggesting that inoculation of PFF causes spreading in anatomically interconnected brain regions (Masuda-Suzukake et al., 2013; Recasens and Dehay, 2014; Paumier et al., 2015).
(3) Injection of PFF into wild-type mice can generate the same results with transgenic mice (Luk et al., 2012a,b; Masuda-Suzukake et al., 2013, 2014; Recasens and Dehay, 2014).
(4) When injecting recombinant monomeric and oligomeric α-syn into the olfactory bulb of wild-type mice, the formation of α-syn aggregates is observed in interconnected regions (Rey et al., 2013; Recasens and Dehay, 2014).
(5) Injection of LB extracts containing α-syn from the brain tissues of post-mortem patients with PD into the striatum or substantia nigra of wild-type mice triggers neurodegeneration and the aggregation of α-syn in nigral neurons and anatomically interconnected areas. However, this was not observed when injecting the LB extract into mice lacking α-syn expression (Recasens and Dehay, 2014; Recasens et al., 2014; Fernandez-Borges et al., 2015; Dehay and Fernagut, 2016).
(6) Intramuscular injection of PFF into the hind limbs of mice expressing either wild-type or A53T mutated α-syn, causes α-syn pathology swiftly in the brain and a rapid-onset motor phenotype. Injection of α-syn fibrils via intravenous and intraperitoneal routes in α-syn-overexpressing mice can also induce the above pathological process. These findings reveal that α-syn can give rise to neuro-invasion from peripheral exposures (Sacino et al., 2014; Ayers et al., 2017).
(7) The significant discoveries of intra-nigral injections of recombinant adeno-associated viral vectors (AAVs), encoding either human wild-type or mutated α-syn, include: (1) the production of cytoplasmic α-syn inclusions and α-syn-loaded dystrophic neurites like PD; (2) the generation of post-translationally modified α-syn; (3) the accumulation of high-molecular-weight α-syn detectable both biochemically and also immunohistochemically; (4) synaptic dysfunction; (5) the reduction of striatal dopamine. In 2013, Ulusoy and colleagues developed a new AAV model that targeted the vagal system, which demonstrated inter-neuronal transmission of α-syn *in vivo* and a direct relationship between α-syn overexpression and efficient CNS protein propagation for the first time. The animal model with vagal injections of α-syn-carrying AAVs is characterized by a caudo-rostral pattern of spread that shares similarities with the pattern of the anatomical distribution of α-syn pathology in PD (Fernandez-Borges et al., 2015; Ulusoy et al., 2015, 2017; Helwig et al., 2016; Recasens et al., 2018).

With time, α-syn aggregates spread through the brain following axonal projections. The strongest evidence for α-syn propagation is the early work where microfluidic devices were utilized, which effectively separates somata from axonal projections in fluidically isolated microenvironments. It was demonstrated that α-syn fibrils are internalized, anterogradely transported within axons, released, and subsequently taken up by additional neurons. The findings by Freundt et al. suggested that transmission can occur in the absence of synapses, because their formation in mixed E17 murine neuron/astrocyte cultures requires 2–3 weeks, whereas the second-order neurons had been cultivated for only 1 or 4 days. If axon-to-somata transmission of misfolded α-syn occurs in a similar pathway in the CNS, the extracellular progresses may offer an opportunity to block fibrillar α-syn (Freundt et al., 2012). Brahic et al. (2016) observed that α-syn fibrils are transported along axons, both in the anterograde and retrograde direction and secreted by axons after anterograde transport, in the absence of axonal lysis, hinting that trans-neuronal transfer can occur in intact healthy neurons. The kinetics of transport suggests that α-syn fibrils are part of the slow component b of axonal transport (Freundt et al., 2012; Bieri et al., 2018). It was verified that accumulation of pathologic α-syn led to selective reduction in synaptic proteins, progressive impairments in neuronal network function, and excitability that all culminate in neuron death. However, data in rodents is still equivocal, as much of the published data can be explained simply via retrograde transport of protein (Taylor et al., 2005; Volpicelli-Daley et al., 2011).

All the experimental findings clearly illustrate that misfolded α-syn is the “spreading agent,” and is characterized by a “prion-like” behavior of transcellular propagation.

There have been recent studies on the methods that allow for monitoring prion-like propagation of the pathogenic protein between diverse cells in the *Drosophila melanogaster* model. This experimental paradigm can be useful for investigating and identifying molecular mechanisms underlying α-syn pathology in PD (McGurk et al., 2015; Donnelly and Pearce, 2018).

#### Cellular Release of α-Synuclein

In 2003, the first evidence that demonstrated α-syn present outside the cells was the discovery of α-syn in cultured neuroblastoma cells, CSF, and plasma, and this was observed earlier than the concept that α-syn would act as prions do. These observations showed multiple molecular species of α-syn in the extracellular space of the human CNS regardless of disease conditions. However, the relevant regulatory mechanism of secretion, the correlation of α-syn aggregation, and their localization are unknown (Tyson et al., 2016).

It is generally believed that α-syn can be transferred by classic exocytosis and endocytosis pathways (including micropinocytosis), tunneling nanotubes (TNTs), synapses or synapse-like structures, and several receptors (Goedert et al., 2010; Dunning et al., 2012; De Cecco and Legname, 2018).

Recent studies indicate that α-syn exists in secretory vesicles of neurons and multifarious biological fluids such as CSF and plasma, implying that α-syn may be secreted via exocytosis (Goedert et al., 2010; Lee et al., 2010; Tyson et al., 2016). There is growing evidence supporting that α-syn could be transferred by two ways. The first is through an exosome-associated mechanism in a calcium-dependent manner, further exacerbated after lysosomal inhibition. Because of their nanometric size, exosomes can pass through the endothelial cells of the blood-brain barrier by receptor-mediated endocytosis. Exosomes are regarded as an indispensable means of transport in CNS. Exosomes are rich in RNA transcripts, especially in small RNAs. When exosomes are ingested by cells, the embedded RNAs can regulate the expression of their target genes. The presence of misfolded α-syn and unique RNA profiles can provide effective biomarkers that favor the diagnosis and treatment of PD (Quek and Hill, 2017). The other mechanism through which α-syn can be transferred is vesicle-mediated exocytosis. Extracellular vesicles containing α-syn are released by primary neurons under stress induced by the lipid peroxidation product 4-hydroxynonenal, internalized into by secondary neurons and trafficked within axons. These results suggest that α-syn transfer via extracellular vesicles may be at least partially involved in the spread of PD (Zhang et al., 2018). More studies hint that the mechanism of release may be non-classical ER-Golgi–independent exocytosis (Kaufman and Diamond, 2013). Moreover, Jang and colleagues demonstrated that inhibition of mitochondrial complex I, as well as proteasomal and lysosomal activities, could affect exocytotic α-syn release (Sian-Hulsmann et al., 2015; Tyson et al., 2016).

Tunneling nanotubes (TNTs) containing F-actin, whose diameter is < 200 nm, are an important mechanism for α-syn propagation in a similar manner as prions. By using quantitative fluorescence microscopy with co-cultured neurons, α-syn fibrils efficiently transfer from donor to acceptor cells through TNTs inside lysosomal vesicles (Abounit et al., 2016a). TNTs allow direct physical connections of remote cell membranes. Theirs physiological role is to transfer cellular components, but under pathological conditions TNTs can facilitate the spreading of viruses and pathogenic proteins (Sherer and Mothes, 2008; Dunning et al., 2012). It is postulated that misfolded protein aggregates can promote the formation of TNTs and thereby their own intercellular transfer, contributing to the propagation of pathology. Because intracellular accumulation of misfolded α-syn induces lysosomal impairments, neurons try to dispose of impaired material through TNTs, which enhances their formation. The accumulation of α-syn in lysosomes may change organelle signaling and positioning, making them have a stronger tendency to enter the new TNTs. Proteolytic degradation of misfolded α-syn in the lysosome prevents neurons from α-syn seeding pathogenicity (Sacino et al., 2017). Moreover, truncated species of α-syn show higher seeding propensity, suggesting that tuning of lysosomal protease activity under different conditions may modulate pathogenicity of the α-syn seeds (Karpowicz et al., 2019). Furthermore, lysosomal impairment can directly or indirectly influence mitochondrial health and function, which may ultimately mediate toxicity in PD. As a result of lysosomal damage, the rate of aggregate growth in neurons increases, suggesting that decreased α-syn seed degradation, increased escape from damaged lysosomes, or both, can potentiate recruitment of α-syn into aggregates (Abounit et al., 2016b; Victoria and Zurzolo, 2017; Karpowicz et al., 2019). Astrocytes, as the major cell type of the brain, may actively transfer α-syn aggregates to healthy astrocytes by direct contact or TNTs. This finding reveals astrocytes as a potential target for PD therapy (Bukoreshtliev et al., 2009; Rostami et al., 2017). Although these structures are known to be present in cultured cells, their formation in tissue is still controversial and uncharacterized.

Another approach for α-syn spreading is necrotic neurons death, which releases intracellular contents. Nonetheless, many studies show that the majority of monomeric α-syn outside the cells originates from active release instead of passive release from neuron death (Costanzo and Zurzolo, 2013).

Subsequently, several studies showed that α-syn may be actively secreted and this process can be constitutive and regulated. In induced pluripotent stem cell (iPSC)-derived cells that express a triplicate of the gene encoding α-syn, high levels of the protein can be found in their extracellular medium. These results indicate that there is an interrelation between the amount of α-syn in the cell and its secretion to the extracellular space. This finding may be applicable during aging in PD, where there are higher levels of cytoplasmic α-syn in neural cells, and therefore greater secretion of α-syn (Woodard et al., 2014; Tyson et al., 2016).

#### Cellular Uptake of α-Synuclein

Following intracortical injection of recombinant α-syn in rats, the cellular uptake was attenuated with co-injection of an endocytosis inhibitor. This fact demonstrates the significant role of endocytosis (Hansen et al., 2011). In primary neurons transduced with recombinant α-syn PFFs labeled with environmentally-responsive fluorophores, the vast majority of PFFs are acidified along the endocytic pathway and remain there for a week or more (Karpowicz et al., 2017). The ability to uptake extracellular α-syn, and subsequent degradation, is in connection with the α-syn species with different dimensions. The entry of larger aggregates requires specific entry pathways, including dynamin-dependent endocytosis. Oligomeric and fibrillar α-syn are taken up by cold temperature and dynamin K44A-sensitive endocytosis (Kaufman and Diamond, 2013; Chan et al., 2017). Diffusion through the cell membrane is one of the simplest pathways for cellular uptake. Monomeric α-syn is capable of readily diffusing through the cell membrane, because its uptake cannot be inhibited by low temperature or blocked as in typical pathways like endocytosis.

Most classical endocytosis requires receptors to mediate internalization. Different studies report the existence of α-syn receptors that may mediate the cell-to-cell transmission of α-syn pathology. The first specific mediator of α-syn was a class of glycosylated extracellular matrix proteins known as heparan sulfate proteoglycans (HSPGs) (Holmes et al., 2013; Karpowicz et al., 2019). HSPGs are capable of mediating macropinocytosis related to α-syn and other aggregation-prone proteins (Holmes et al., 2013; Stopschinski et al., 2018). The α3 subunit of the Na^+^/K^+^-ATPase can interact with both oligomeric and fibrillar α-syn at the cell surface. The Fc gamma receptor IIb is described as a receptor for α-syn fibrils. The lymphocyte-activation gene 3 (LAG3) binds misfolded α-syn built-in in PFFs with high selectivity but does not interact with monomeric α-synuclein (Mao et al., 2016). The proposed pathway for LAG3-dependent uptake is clathrin-mediated endocytosis (CME), which may participate in α-syn monomer uptake (Konno et al., 2012; Karpowicz et al., 2019). Similar specificity to aggregated, but not monomeric α-synuclein, is described for toll-like receptor-2 (TLR2) and transmembrane ion channels receptor P2X7. Neurexin 1α is described as a cell surface receptor for both α-syn PFFs and fibrils. α-syn fibrils were also shown to interact with amyloid-β precursor-like protein 1 and heparin sulfate proteoglycans that mediate endocytosis. Interestingly, PrP^C^ is reported as a possible receptor for α-syn amyloid fibrils, facilitating their internalization through and endocytic pathway (Urrea et al., 2017; De Cecco and Legname, 2018). The *in vitro* data confirmed that the presence of PrP^C^ facilitates the higher and faster uptake of α-syn fibrils, which was also shown *in vivo* in wild type (*Prnp*+/+) compared to PrP knock-out (*Prnp*-/-) mice (Aulic et al., 2017; Urrea et al., 2018). This cooperation fosters the transmission of α-syn between cells and causes synaptic dysfunction via a signaling cascade acting through phosphorylation of Fyn kinase and activation of the N-methyl-D-aspartate receptor (NMDAR). α-syn-PrP^C^ binding induces cofilin/actin rods formation, which changes actin dynamics, resulting in rearrangements of cytoskeleton and eventual synaptic dysfunction (Clavaguera et al., 2015; Bras et al., 2018; Surguchev et al., 2019). Equally, α-syn amyloids blocked the replication of PrP^Sc^ *in vitro* and *ex vivo* (Aulic et al., 2017). Deciphering the mechanisms involved in sensing diverse forms of extracellular α-syn may prove invaluable in our quest to devise novel diagnostic and therapeutic approaches in PD. The internalized fibrils and oligomeric α-syn are trafficked via the endosomal pathway and degraded by the lysosome. In contrast, the monomer of α-syn rapidly pass through the plasma membrane before being degraded by the proteolytic systems (Lee et al., 2008; Chan et al., 2017).

**FIGURE 2:**
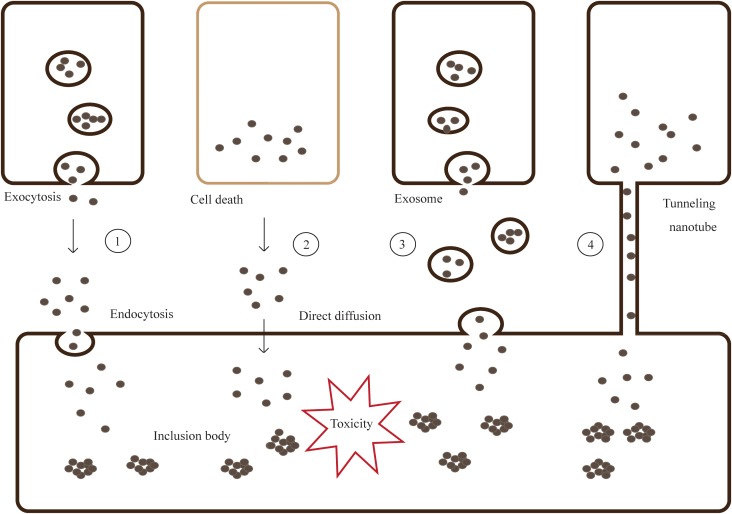
Models for cell-to-cell transmission of misfolded and aggregated proteins. Step 1- Misfolded and aggregated proteins are released from neurons by vesicle-mediated exocytosis, then taken into the neighboring cells via endocytosis. Step 2- Proteins are spilled from dead cells and enter the neighboring cells through direct diffusion. Step 3,4- Proteins can transmit by packaging into exosomes or tunneling nanotubes (TNTs).

Introduction of α-syn aggregates by PFFs generated from truncated recombinant human wild-type α-syn into primary hippocampal neurons, cause adsorptive-mediated endocytosis promoting soluble α-syn into insoluble LBs and LNs (Volpicelli-Daley et al., 2011; Jellinger, 2012). When a GTPase-deficient Rab5A was introduced into the cells, α-syn uptake and cell degeneration decreased. This indicates that α-syn could be taken up through Rab5A-dependent endocytosis (Tyson et al., 2016) (Figure 2).

Ultimately, internalized α-syn proteins act as amplification seeds by recruiting endogenous native proteins and leading to formation of toxic LBs.

There are key factors that dramatically modulate the prion-like propensities of α-syn, including the concentration of nuclei, the presence of oligomers, and the toxicity, resistance, and localization of α-syn aggregates. In brief, these mentioned factors which favor the high concentration of extracellular nuclei or oligomers, characterized by small size, with a low toxicity would effectively enhance prion propensity; in contrast, low concentrations of highly toxic intracellular aggregates, with a larger size, would obviously prevent spreading (Espargaro et al., 2016).

In addition to α-syn aggregation, inflammation also plays a key role in the progression of PD. Chemical or viral exposure may give rise to immune activation in the gastrointestinal tract or olfactory system, triggering misfolding, aggregation, and subsequent propagation of α-syn. The dysregulated action of glial cells and astrocytes triggered by the neurotoxic α-syn can induce inflammation in the brain. It is proposed that neuroinflammation can prompt the prion-like pathology of α-syn by increasing its release, uptake, or both. But this hypothesis has not been fully confirmed. However, further studies about the relation between inflammation and prion-like behavior is required to support the hypothesis (Lema Tome et al., 2013; Lawand et al., 2015; Chandra et al., 2017).

## Implications for Therapeutic Strategies

There is no cure for PD and available palliative treatments only focus on restoring dopamine deficits and controlling the symptoms. Present efforts for therapies of PD focus mainly on restraint for the templated conformation change and transcellular propagation. The former includes stabilizing the physiological conformation of synuclein, decreasing its expression, preventing aggregation of α-syn, and increasing intracellular clearance of the aggregates; and the latter consists of preventing the release, decreasing uptake by cells, and increasing extracellular clearance (Kaufman and Diamond, 2013; Hasegawa et al., 2017). In view of the normal physiological function of α-syn, Benskey and colleagues proposed the hypothesis that loss of a-syn function within nigrostriatal neurons initiates a neuronal-mediated neuroinflammatory cascade, ultimately resulting in the death of affected dopaminergic neurons. Therefore, more studies are needed for reasonable intervention of α-syn clearance (Benskey et al., 2018). Beyond that, anti-inflammation approaches may be used as new therapeutic methods. Considerable research is currently directed on targeting proinflammatory mediators, such as cytokines and the transcription factors that regulate their expression, trying to identify a novel treatment of PD (Lawand et al., 2015). Small molecular compounds that are capable of blocking aggregation of intracellular α-syn have remarkable application prospects for pharmacotherapy of PD. These compounds firstly enter the brain, then specifically bind to the abnormal α-syn and inhibits its action (Lawand et al., 2015; Hasegawa et al., 2017).

For the treatment of PD, the most effective therapy to inhibit the propagation of α-syn is knock-out of the related gene or inhibiting protein expression via gene or RNAi therapy. However, as mentioned earlier, it is still unknown whether gene therapy will affect α-syn normal function. Further studies are needed to evaluate the relevant efficacy before the approach can be used for clinical treatment (Kaufman and Diamond, 2013; Nielsen and Nielsen, 2013; Braak and Del Tredici, 2016; Hasegawa et al., 2017).

Furthermore, immunotherapy targeting extracellular α-syn is showing great promise. Experiments in PD models indicate that active vaccination against recombinant α-syn leads to ameliorated pathology (Sian-Hulsmann et al., 2015). Similarly, passive immunotherapy also produced positive effects. Antibodies produced by immunotherapy are expected to block the spreading of pathological α-syn (Lawand et al., 2015). Moreover, the spreading of α-syn in wild-type mice after intracerebral inoculation of α-syn fibrils was prevented by monoclonal antibodies (mAbs) against abnormal α-syn. α-Syn mAbs reduce α-syn PFF-induced LBs/LNs formation and rescue synapse/neuron loss in primary neuronal cultures by preventing both uptake and subsequent cell-to-cell transmission of pathology (Tran et al., 2014).

Existing symptomatic therapeutic strategies for treating PD include L-DOPA and monoamine oxidase (MAO) inhibitors. On the other hand, more attention has been paid to the experimental disease modifying treatments such as neurotrophic factors, neuroprotective factors and stem cell regeneration. Neurotrophic factors are a family of secreted proteins that participate in neuronal survival and neuroplasticity. These proteins can be upregulated together with their receptors under pathogenic conditions which corresponds with the notion that they are protective and enhance brain plasticity, thus avoiding brain damage (Ibanez and Andressoo, 2017). One of the most studied is glial cell line-derived neurotrophic factor (GDNF), which has neuroprotective and neurorestorative effects in PD animal models, however, it is challenging to achieve such neuroprotection in clinical trials on PD patients (Francardo et al., 2017). Another widely studied neuroprotective factor is Brain-derived neurotrophic factor (BDNF), which is a ubiquitous neurotrophin in the adult brain, maintaining dopaminergic neuronal survival, promoting synaptic plasticity, dendritic morphogenesis and arborization, and even neurogenesis (Rahmani et al., 2019). Moreover, α-syn effectively blocks neurotrophic activity of BDNF in SN, first by downregulating BDNF expression (Yuan et al., 2010), and second by competitive inhibition of BDNF signaling at receptor level (Kang et al., 2017). Most studies have a consensus that exogenous introduction of BDNF is able to mitigate dopaminergic neuronal loss in neuronal culture and in animal models of PD (Sadan et al., 2009; Zhao et al., 2014; Goldberg et al., 2015). It was also reported that anti-PD drugs, even dopamine replacement treatments, performed part of functions by upregulating BDNF (Rahmani et al., 2019). Another treatment strategy is DA neuron restoration in damaged regions by neurogenesis stimulation or iPSCs. One way of stimulating neurogenesis is through the activation of the transcription factors, such as nuclear receptor related 1 (Nurr1) (Kim et al., 2015). Furthermore, a PD animal model with iPSC-derived neuronal stem cells transplanted into the striatum showed improvement in functional defects of rotational asymmetry. Moreover, the neuronal stem cells survived and integrated into the brains of transplanted PD animals and differentiated into neurons, including DA neurons. Clinical application of the experimental treatments for PD may become an attractive strategy in the future (Han et al., 2015; Van Bulck et al., 2019).

However, these treatments rely on discriminating the correct α-syn species for intervention and a deeper understanding of the interrelation between the degradation and propagation of α-syn. Because of the variety of pathogeneses, combination therapies receive increasing attention compared to single-target agents (Valera and Masliah, 2016). Further studies on underlying influencing factors are expected to reveal the best targets for the treatment for PD.

Although the inoculation of α-syn aggregates may induce neuronal damage and clinical abnormalities (e.g., motor impairments) in wild-type mice, none of available studies provided evidence for a transmission of PD between individuals. The findings published so far on the effects of experimentally transmitted α-syn seeds do not indicate specific precautionary measures in the context of hemotherapy, but call for vigilance in transfusion medicine and other medical areas. This suggests that patients may possibly get medical security by a thorough decontamination from the α-syn aggregates in surgical instruments, blood or blood products and medical services (Beekes et al., 2014; Recasens et al., 2014).

A novel real-time quaking-induced conversion (RT-QuIC) based assay is able to detect α-syn aggregation in brain and CSF from PD patients with a sensitivity of 95%, and with a specificity of 100% (Le et al., 2015; Fairfoul et al., 2016). It is demonstrated that α-syn can be rapidly detected and quantitated, even in early symptomatic stages of synucleinopathy (Groveman et al., 2018). By this token, RT-QuiC analysis of CSF is potentially useful for the early clinical assessment of PD patients (Fairfoul et al., 2016).

Several diagnostic imaging probes were reported to monitor cerebral amyloid lesions in neurodegenerative disorders. Florbetapir is the first radioactive dye for brain imaging of amyloid plaques approved by the FDA in AD. The importance of further studies for their practical implications in therapy and diagnostics should be highlighted (Aulic et al., 2013).

## Dissenting Opinions

In this review we described the prion-like hypothesis in PD from a biophysical point of view and discussed the literature in support of the hypothesis. Pathological α-syn is known as prion-like or prionoid, which describes the dominating properties that PrP^Sc^ and pathological α-syn have in common: seeding/templating, propagation, structurally differentiated conformations, and subsequent neurodegeneration. While it is evident that PrP^Sc^ and pathological α-syn share properties, not all experimental observations can be entirely explained by this fact. The most significant difference between pathological α-syn and PrP^Sc^ is that PrP^Sc^ is transmissible between humans and PD-associated α-syn is not. However, human-to-human transmission of prion diseases is nowadays restricted to iatrogenic incidents. The presence of amyloid-β protein (Aβ) deposits have been reported in patients with CJD who had been treated with human cadaveric pituitary derived growth hormone (c-hGH) contaminated with prions during childhood with short stature. Purro et al. verified the presence of Aβ seeds in archived c-hGH vials and proposed a hypothesis about the iatrogenic human transmission of Aβ pathology. Doubts should be raised regarding whether transmission of α-syn may occur under special circumstances (Purro et al., 2018).

In terms of the potential propagation of α-syn, the staging for PD is an indirect measure. All information on α-syn propagation are rooted in experimental animals, primarily rodents, but not from humans. Another point of difference is that PD is not rapidly progressive like prions. Neurodegenerative diseases are generally considered to be chronic diseases, and the rate of disease progression is also one of their differences.

## Conclusion

It is believed that self-aggregation and transmission of α-syn contribute to the gradual spreading of pathological degeneration in PD. Misfolded aggregates escape from clearance and are released into the extracellular space via synapse, exosome, vesicle,cell death, or tunneling nanotubes. Afterward, α-syn aggregates are internalized by the recipient cells and induce endogenous α-syn to misfold and assemble in some unclear manner. All the studies mentioned in this review support the fact that misfolded α-syn have much in common with prions in their mode of action: (i) α-syn and prions both mainly exist either without a defined structure or in an α-helix, however, they tend to misfold when the conformation is rich in β-sheets under certain circumstances. (ii) Misfolded proteins lead to further accumulation of normal proteins and acts as template to change their conformation. (iii) Misfolded proteins are capable of being taken in by and transferred from cell to cell. However, there are still plenty of unanswered questions regarding the pathogenesis of α-syn: (i) the specific molecular mechanisms of misfolding, release, uptake, and transmission of the aggregates; (ii) whether the misfolded protein in a cell can induce protein misfolding in the neighboring cells directly; (iii) the physicochemical and environmental factors that affect the pathological process; (iv) whether the diversity of species and conformations has an impact on clinical manifestations; and (v) whether PD has the same infectivity as prion diseases. By asking these questions, we are witnessing the beginning of a new field of research in PD and other neurodegenerative disorders.

## Author Contributions

JW and AX planned the study. JM, JG, and JW analyzed the data and edited the manuscript. JM wrote the manuscript.

## Conflict of Interest Statement

The authors declare that the research was conducted in the absence of any commercial or financial relationships that could be construed as a potential conflict of interest.
